# Structural analysis of social behavior: Using cluster analysis to examine personality profile associated with diabetes onset

**DOI:** 10.1371/journal.pone.0315895

**Published:** 2025-05-09

**Authors:** Anna Vespa, Roberta Spatuzzi, Paolo Fabbietti, Mirko Di Rosa, Maria Paola Luconi, Giorgio Arnaldi, Giancarlo Balercia, Gianmaria Salvo, Riccardo Sarzani, Maria Velia Giulietti

**Affiliations:** 1 Scientific and Technological Area, Department of Neurology, INRCA-IRCCS National Institute of Health and Science on Aging, Ancona, Italy; 2 Department of Mental Health, ASP Basilicata, Potenza, Italy; 3 Biostatistical Center, INRCA-IRCCS National Institute of Science and Health on Aging, Ancona, Italy; 4 Centre for Biostatistics and Applied Geriatric Clinical Epidemiology, IRCCS INRCA, Ancona, Italy; 5 Diabetology Unit, National Institute of Health and Science on Aging, IRCCS INRCA, Ancona, Italy; 6 Department of Endocrinology, Policlinico “P. Giaccone”, Palermo, Italy; 7 Division of Endocrinology, Department of Clinical and Molecular Sciences, Polytechnic University of Marche, Ancona, Italy; 8 Hypertension Excellence Centre ESH, Polytechnic University of Marche, Ancona, Italy; Universita Politecnica delle Marche, ITALY

## Abstract

**Objective:**

In this study we tested whether some intrapsychic behaviors of structure of personality can be associated with the onset of Type 2 diabetes (T2-DM).

**Methods:**

T2-DM Patients (n. 257) and Healthy subjects (n = 258). Test: Social schedule (demographic variables); SASB–Form- Questionnaire A (describing intrapsychic behaviors of personality structure - 8 Clusters-Cl -).

**Results:**

From the logistic model emerged that in subjects with profile 2 “Low Affiliation and Autonomy” compared to profile 2 “Low Autonomy and Self-Care” considering age, education and living conditions effects, the association to the onset of diabetes increases (OR: 1.668). Subjects with profile 2 “Low Affiliation and Autonomy” show low assertiveness and autonomy (SASB-Cl 1); have a medium-low ability to accept and support themselves (medium-low-SASB-Cl 3,4); do not improve their leisure activities or interpersonal relationships because they are too scheduled by things to do (SASB-Cl 4, Cl5); have self-critical behavior (SASB-Cl 6 medium) and self-neglectful behavior - ignore illnesses at an emotional and physical level (Cl8). They occasionally incur in self-destructive behaviors (Cl 7). They may experience self-exhaustion and mild to moderate depression. These are the intrapsychic behaviors associated with onset of diabetes. Another variable associated with the onset of diabetes is high educational level.

**Conclusions:**

These results suggest that certain personality traits have a great association with T2-DM onset. Furthermore the analysis of intrapsychic modalities in association with the onset of diabetes could constitute a further screening aimed at primary prevention.

## Introduction

Type 2 diabetes mellitus (T2-DM) is an increasingly common chronic disease, which requires those who are affected to undergo a lifelong therapeutic self-management regime. It is estimated that by 2045, the percentage of adults with type 2 diabetes (which constitutes 90% of this disease) in the world population will increase from about 10% to 12.2%, without considering undiagnosed diabetes [1]. Diabetes is associated with severe complications such as kidney failure, lower limb amputation, blindness, and cardiovascular diseases, which compromise individuals’ functional capacity, autonomy, and quality of life complications are often serious and the mortality rate is very high [1–4].

There is no known cure for diabetes. The adherence to self-management regime, linked to the subjects’ emotional and cognitive abilities, is influenced by the patients’ personalities [5].

Some studies have pointed to some personality factors associated with poor restraint as determinants of ill health [6–9]. The Type D personality that refers to the combination of negative affectivity, tendency to experience negative emotions and social inhibition (tendency to inhibit self-expression, poor social adaptation, e.g., poor positive affection, aggression, shyness, worry and pessimism, lack of flexibility, resistance to change) [10,11] has been linked to poor health, morbidity and mortality in different diseases such as cardiovascular diseases [7–9]. This personality is characterized by a lowered resistance to stressful events leading to the hyperactivation of autonomic systems with reduced immunity [4,6,7,11].

The subjects with Type D personality may avoid expressing their emotions in social interactions due to fear of social rejection or any other kind of disapproval from others: they may have a perceived lack of social support [8]. These behaviors were significantly associated with depressive symptoms [6–12]. A potential mechanism by which the Type D personality can exert a negative influence on health is non-optimal self-care behavior [12–14]. Thus, the Type-D distressed personality factor affects the main clinical variables of diabetes such as glycemic control and self-management, adherence to drug therapy and treatment compliance, medical consequences and complications, diet and lifestyle, and healthy behaviors [6,7,13,15–20]. While the main therapeutic goal in diabetes is medical adherence, it is important to clinically detect the effects of personality traits on diabetes prognosis [5,8,16,17] and the individual ability to cope with stress.

Stress is the response of the whole organism to challenging stimuli (stressors) that induces physiological, behavioral, and psychological adjustments to cope with situations.

Indeed, dealing with diabetes management increases the risk of developing subclinical or clinical depression due to changes in lifestyle and health behavior, as well as endocrine changes; on the other hand, another study adds evidence that depression is associated with a higher risk of developing T2DM, and the association is stronger among people with severe depression [18–29]. The direction of the association is likely to be bidirectional

However it is not only the presence of negative emotions that should be considered as a risk factor for health but also how an individual deals with these negative emotions and in this direction the personality plays an important role [5–7,19,27–31].

In fact, personality is a potentially relevant factor that has received relatively little attention, even though extensive longitudinal studies have shown that personality predicts a number of aspects of ill health and longevity [3,4,8,10,12,16,31].

The question that arises is whether the personality characteristics described in diabetic patients may pre-exist and be associated with the onset of diabetes, precisely because they are connected to the coping mechanisms of stress and to the individual lifestyle [28,31–35].

Inter-individual differences in health self-management are fundamental in adapting to the disease condition, but, also in maintaining health [34,35].

Knowing these differences in personality and style of life may be important not only for providing effective support to sick people but it could also prevent them from getting sick.

There are studies on sociodemographic variables, i.e., age and educational level, and lifestyle-related risk factors for identifying vulnerable groups for Type 2 diabetes. [35,36].

There are many studies on unhealthy behaviors of the style of life of Type-2 diabetes such as incorrect diet, low physical activity, which are linked to psychological factors but, to our knowledge, no studies on personality traits as risk factor for diabetes onset were found [35].

Furthermore, to our knowledge, there are few studies that used a combinations of traits [37] rather than single personality traits (e.g., neuroticism), to identify personality dimensions that may increase the risk of depressive, anxious, and physical symptoms among patients undergoing treatment for Type 2 diabetes, and no studies on personality traits associated with the onset of diabetes [33,35,36].

On the other hand, many studies have shown that cluster analysis allows to identify latent classes (subgroups) of individuals with distinct profiles of personality dimensions considering a wide range of personality characteristics. [37–39] In this context, knowledge of intrapsychic behaviors allows to better understand this issue [39–41].

Furthermore, the cluster analysis, by providing an accurate description of intrapsychic processes, could confirm and/or deepen and clarify some aspects of the type D personality. Therefore, having a good understanding of such psychological dynamics may contribute to implement primary and secondary preventive treatments and improve the quality of life and survival in patients with T2-DM [15,42].

For this reason, in this study we implemented the Structural Analysis of Social Behavior - SASB model by L. S. Benjamin [38–41]. which allows to describe how intrapsychic processes are experienced and their changes based on interpersonal experiences, considering the totality of the person and not limited to pathological characteristics. In fact, this model does not neglect normal interpersonal functions and processes, which are considered fundamental from the point of view of theoretical explanation and planning and verification of change processes.

Thus, the SASB Model can be used as a diagnostic and programming tool for interventions aimed at promoting growth and maturation intrapsychic structures.

According to L. S. Benjamin, the intrapsychic and interpersonal SASB characteristics of personality could be associated with specific somatic diseases.

The SASB model includes three underlying dimensions for the description of both interpersonal and intrapsychic experiences: focus (other, self, introject), affiliation and interdependence.

The assessment of personality profiles of Type 2 diabetic patients and healthy subjects could be useful in subgrouping them to identify vulnerable individuals and to ensure tailored interventions [4,6,31,42]. We hypothesize that some intrapsychic behaviors (SASB), also described by Type D personality, such as poor self-care and poor restraint, due to low autonomy and spontaneity, neglect of one’s own needs at an emotional and physical level, self-criticism, which might translate into a specific expected profile may not only be experienced by diabetic patients but may be factors associated with the onset of diabetes.

At this point the question that arises is the following: could personality structure influence the onset of type 2 diabetes? Are there intrapsychic and interpersonal behaviors (traits) of the personality structure that determine the individual lifestyle and the effective or ineffective coping with stressful situations that can promote the onset of T2-DM? In the present study we examined this topic.

## Materials and methods

### Study population

#### Participants.

Two hundred fifty-seven Type-2 diabetic patients (DP) (Table 1) were enrolled by the diabetologists from the Department of Diabetology (INRCA-IRCCS) and in Endocrinology Department of Union of Hospital, Polytechnic University of Marche in Ancona (Italy). Type 2 diabetes diagnosis was made according to the American Diabetes Association Criteria [43].

**Table 1:** Description of the sample.

	Healthy Subjects	Diabetic Patients	Total	P value
	(n = 258)	(n = 257)	(n = 515)	
Age	54.0 ± 11.8	55.7 ± 14.1	54.9 ± 13.0	0.145
Sex, female	138 (53.5)	143 (55.6)	281 (54.6)	0.624
Live Alone, Yes	36 (14.0%)	27 (10.5%)	63 (12.2%)	0.233
Educational level:				0.108
3° Elementary	8 (3.1%)	1 (0.4%)	9 (2.7%)	
5° Elementary	126 (48.8%)	117 (45.5%)	243 (47.2%)	
Secondary School	55 (21.3%)	56 (21.8%)	111 (21.6%)	
High School	41 (15.9%)	54 (21.0%)	95 (18.4%)	
University degree	28 (10.9%)	29 (11.3%)	57 (11.1%)	
SASB1	3.9 ± 1.3	4.3 ± 1.2	4.1 ± 1.3	<0.001
SASB2	6.0 ± 1.8	6.5 ± 1.5	6.3 ± 1.6	0.001
SASB3	5.9 ± 1.7	6.4 ± 1.5	6.1 ± 1.6	0.002
SASB4	5.7 ± 1.5	6.4 ± 1.5	6.0 ± 1.5	<0.001
SASB5	5.3 ± 1.5	5.4 ± 1.3	5.4 ± 1.4	0.148
SASB6	1.7 ± 1.7	1.5 ± 1.7	1.6 ± 1.7	0.170
SASB7	1.6 ± 1.6	1.3 ± 1.5	1.5 ± 1.6	0.018
SASB8	2.3 ± 1.7	2.1 ± 1.6	2.2 ± 1.7	0.236

The patients were affected by type 2 diabetes from 1 to 9 years, aged between forty and seventy-five (45% were aged between forty and fifty-nine and 55% between sixty and seventy-five).

Exclusion criteria. Clinical evidence of cardiovascular disease CVD evaluated as follow: ictus by clinical history; ischemic heart disease and acute myocardial infarction by clinical history and by resting electrocardiogram; peripheral vascular disease by clinical history and, for lower limbs, by ankle-brachial index cancer (e.g., patients with colorectal cancer, tumor necrosis); neurological disease (e.g., patients with Parkinson, multiple sclerosis), and other different medical disorders (i.e., fibromyalgia, irritable bowel syndrome, patients with renal disease, or patients suffering from asthma).

The healthy group (CG) was composed of two hundred fifty-eight healthy subjects (without diabetes and clinical history of CVD or other diseases). These subjects were chosen on the basis of the same demographic variables of the study groups (age, civil status and cultural level) (Table 1).

The study protocol was approved by the Bioethics Advisory Committee IRCCS-INRCA, Ancona, Italy; code no. 16025. Sampling for the study began on 21/05/2018 and was discontinued due to COVID lock-down. It was resumed on 15/04/2022 and completed on 22/07/2023.

All participants signed a consensus form regarding the study protocol after detailed explanation by the physician in the Clinics. Three hundred and thirty-nine diabetes patients were approached in the clinic by the physician and asked to participate in the study. Only three hundred and eleven patients decided to participate and to fill out and sign the consent form. The questionnaire was administered to patients in the context of an individual interview conducted by psychologists trained to use the SASB model. The same procedure was followed for the healthy group.

Fifty-tree diabetic patients didn’t answer all the questions in the questionnaires or not meet the inclusion criteria: it was therefore decided not to consider them for the analysis. So the sample is of two hundred fifty-seven diabetic patients.

All diabetic and healthy subjects were asked to complete the following psychological and psychosocial tests:

1. Social schedule, including data on gender, age, marital status, educational level.
2. The SASB – Form-A Questionnaire [39–41], that describes the psychic processes of the personality structure at the intrapsychic level (S1 Appendix). This test was chosen because it is short and has the appropriate reliability and validity to evaluate intrapsychic dimensions from normal to pathological. It is also validated on the basis of DSMIV. This Italian version has been validated on the Italian population. Interviewed subjects had to respond to 36 items in the questionnaire describing their intrapsychic behaviors during the last year (e.g., ‘I neglect myself, don’t try to develop good skills, ways of being’; ‘I practice and work on developing worthwhile skills, ways of being’; ‘I think up ways to hurt and destroy myself. I am my own worst enemy’). They are rated on a 10-point scale ranging from 0 (Never) to 10 (All the time).

The 36 questions of Form-A are grouped by a specific score correction in 8 clusters (Cl) of intrapsychic “Oneself” and interpersonal “Other” experience [41].

The 36 questions of the questionnaire provide an exhaustive picture of intrapsychic experience from which the interpersonal experience can inferred.

The 8 clusters of “Oneself” and “Other” are both complementary and opposite.

The 8 clusters of “Oneself” - Intrapsychic experience - are the following:

Intrapsychic experience: Description of the 8 clusters of “Oneself”

SASB-Cl 1 = Autonomy - Assertive and separating. This type is based on what he/she considers necessary at the time. The attitude may be spontaneous with self-acceptance and pleasure in his/her experience or it could be disoriented with little weight given to problems and important choices in life.

SASB-Cl 2 = Autonomy and love - Self-accepting and exploring. This type of person accepts and reacts to his/her deepest feelings, feeling solid, integrated and “together”. The desire to be open to feelings generally indicates a state of self-satisfaction and acceptance of weak and strong points.

SASB-Cl 3 = Love - Self-supporting and appreciative. This type is deeply appreciative of him/herself and recognizes the ability to treat, care, console and reconsolidate him/herself. It underlines the capacity for self-esteem and in extreme cases, of self-adoration.

SASB-Cl 4 = Love and control - Self-care and development. This type protects and realistically examines the capacity of being positively self-constructive, actively developing his/her abilities and other important qualities for self-growth. This cluster highlights the use of energy to obtain what is needed and desired.

SASB-Cl 5 = Control - Self-regulating and controlling. This type practises self-control. Great self-control is practised for chosen objectives. This may include paying attention to behaviour in order to ensure conforming to ideals, including great activity programmed in order to reach objectives.

SASB-Cl 6 = Control and hate - Self-critical and oppressive. This type of cluster identifies someone who oppresses him/herself and accuses him/herself of inadequacy, evoking feelings of self-guilt and shame. Feelings of uncertainty and guilt can be used by false induction preventing recognition of what is useful and good for the person. This could be self-punitive behaviour, sometimes destructive enough to require therapeutic intervention.

SASB-Cl 7 = Hate - Self-refusing and annulling. This is a self-destructive type who ignores illnesses and wounds, and self-exhaustion. This cluster is implicative of self-refusal and self-deprivation as well as self-inflicted cruelty. Such self-destructive behavior calls for serious qualified psychotherapeutic intervention.

SASB-Cl 8 = Hate and autonomy - Self-negligent and mentally absent. This cluster identifies someone who may daydream, subsequently not developing his/her abilities and potentials to their full extent. In extreme cases, these individuals are unreasonable and have unjustified ideas, regarding behaviour without any criterion and falling into self-destructive situations. In these cases, it could be beneficial to examine the danger of self-destructive behaviour with a therapist.

### Statistical analysis

Demographic and all psychometric characteristics were expressed by mean ± standard deviation for continuous variables and by number and percentage for categorical variables. We compared patients grouped according to diabetes (presence of diabetes vs no diabetes). The chi-square test was used for categorical variables, and ANOVA one-way for continuous ones. Then, a k-means cluster analysis was applied to evaluate the descriptive dimensions of the SASB test from which we obtained 2 profiles. The K-means method seemed very appropriate for this objective and required little time to calculate distances between data points and centroids. The algorithm consisted of three steps:

- initialization: the input parameters were defined;
- cluster assignment (centroid): each point was assigned to the closest cluster;
- update of the centroid position: recalculated the exact point of the centroid and modified its position.”

We included profiles, age, gender and the other variables significant at the univariate level, in a binary logistic model, in order to obtain an adjusted estimate of the relation with diabetes. For all tests, p<=0.05 was considered statistically significant.

Statistical analysis was carried out using SPSS for Win V24.0 (SPSS Inc, Chicago, IL, USA).

## Results and discussion

A total of 257 Type-2 diabetic patients participated in the study. The mean ag 55.7(± 14.1) years. Most of them had primary education (45.9%) (Table 1). The healthy group included 258 subjects with a mean age 54.0(± 11.8). No significant differences in demographic characteristics between the two groups of healthy and diabetic subjects emerged (Table 1).

At the beginning we proceeded with the Clusters analysis of SASB-Form-A- intrapsychic traits of personality. From the comparison between diabetic and healthy groups the following significant differences in SASB clusters emerged: SASB-Cl 1 (p < .001), SASB-Cl 2(p < .001), SASB-Cl 3(p = .002), SASB-Cl 4 (p < .001) and SASB Cl7(p = .018).

Using the K means method 2 SASB profiles were created to identify which may be a predictor for diabetes onset: “Low Affiliation & Autonomy” and “Low Autonomy & Self-Care”. From the comparison of the two profiles, the following significant differences emerged: SASB-Cl 1 Autonomy - Assertive and separating (F = 17.749, p < 0.001); SASB-Cl 2 Autonomy and love - Self-accepting and exploring (F = 331.833, p < 0.001); SASB-CL 3 Love - Self-supporting and appreciative (F = 324.593 p < 0.001); SASB-Cl 4 Love and control (F = 83.120, p < 0.001); SASB-Cl 6 Control and hate - Self-critical and oppressive (F = 327.835 p < 0.001); SASB-Cl 7 Hate - Self-refusing and annulling, (F = 313.956, p < 0.001); SASB-Cl 8 Hate and autonomy - Self-negligent and mentally absent (F = 194.273, p < 0.001). No difference emerged in SASB-Cl5 (S1 Table).

The intrapsychic behaviors were found to be associated with the onset of diabetes: subjects display low assertiveness and autonomy (SASB-Cl1); they have a medium-low capacity for self-acceptance and self-support (SASB-Cl 2,3,4); they do not enhance their leisure activities or interpersonal relationships because they are too programmed by things to do and by self-critical behaviors (SASB-Cl6); they occasionally engage in self-critical (SASB-Cl6) and self-destructive (SASB-Cl7) behaviors; they may incur in self-exhaustion; they have self-neglectful behaviors (ignoring emotional and physical needs) (Cl8); mild to moderate depression is present.

### Profile of personality - logistic regression analysis

From the logistic regression analysis emerged that profile 2“Low Affiliation & Autonomy” compared to profile 1 “Low Autonomy & Self-Care “is associated to the onset of diabetes OR = 1.668), considering the effect of the other variables (Table 2).

**Table 2:** Multiple logistic regression analysis in study groups.

		B	OR		95% C.I.	
				p	Lower	Upper
	Age (years)	0,016	1,016	0,043	1,000	1,031
	Gender (female)	0,102	1,108	0,578	0,773	1,587
	Lives alone	-0,304	0,738	0,287	0,421	1,292
	Educational Level (high school/ university)	0,456	1,579	0,035	1,032	2,415
	Low Affiliation & Autonomy	0,511	1,668	0,016	1,101	2,525
	Constant	-0,895	0,409	0,064		

Also a high level of education is associated with the onset of diabetes (OR = 1.579), always considering the effect of the other variables. Using the Omnibus Test of model coefficients and Hosmer and Lemeshow Test (respectively p = 0.018 and p = 0.043) the overall model equation was significant, and the percentage of explained variance was good (Cox & Snell R square = 0.350).

Our results showed that high level of education is associated with a 57.9% of developing diabetes. The results in this topic are controversial and vary in different populations. According to Xianwen Shang [44] “the subjects with low educational level had a higher risk of diabetes” and Kautzky-Willer [45] states that “the association between education and the chronic diabetes may be of greater magnitude in women: the odds of suffering from diabetes decreased gradually with increasing educational level”. Another author affirms that low educational level had no effect on T2-DM diagnosis rates. [31]

However, our results showing that high educational level of Italian subjects was more associated with diabetes onset could be explained by a richer lifestyle and diet in subjects with higher educational level and we think that the cultural habits of different countries may influence this result [46]. There are many factors, often interrelated, that may have a weight in this contest. Further studies comparing various nations and living conditions can clarify this topic. Furthermore, from our study it emerged that certain intrapsychic traits of personality were associated with the onset of diabetes. This study, to our knowledge, is the first to investigate the association between intrapsychic personality profile and T2-DM onset. It is significant that the “Low Affiliation & Autonomy” personality profile is associated with the onset of T2-DM.

The “Low Affiliation & Autonomy” profile includes intrapsychic behaviors related to low autonomy and freedom (autonomy Cl1), with weak goals in personal choices (neglect of one’s own needs - Cl8). In fact, these people are likely not to carry out their real needs but act to their detriment due to the medium low spontaneity and autonomy (Cl1) in their choice: this may imply using too much energy in carrying out what they have or are used to doing. For example they may neglect and sacrifice leisure time activities to work and/ or commitment activities for family or social duties, such as internal and external demand. These subjects are conditioned by self-imposed goals a priori. People with this profile are inflexible and show resistance to change (Cl1, Cl8). They have a medium-low ability to be positively self-constructive, actively developing skills and other important qualities for personal growth (SASB Cl3, Cl4). They are engaged in behaviors of neglect of their emotional and physical needs even in interpersonal relationships (Cl1, Cl4, Cl8), also because they are not always in touch with their deep feelings, emotions and needs and/or may have difficulties in expressing them (Cl2 and Cl3 medium-low). They may occasionally adopt self-critical behaviors (Cl6). On the other hand, even if they tend to neglect needs and potential for personal growth (Cl4, Cl8), they can only occasionally engage in self-destructive behavior (Cl7). Due to a medium low ability to treat, care, console and reconsolidate themselves, they may have difficulties in taking care of themselves especially when facing stressful events. In fact this neglect can occur especially in the presence of stressful situations.

These people are anxious and tend to be depressed: results in the SASB-Model Form-A questionnaire indicate that patients can experience a state of mild to moderate depression without reaching a level of major depression, as shown by the distribution of ranges in the different clusters.

The SASB model allows us to extrapolate interpersonal experiences from intrapsychic experiences.

Therefore, we will describe the interpersonal experience of subjects with this personality profile.

These subjects do not fully promote independence in their relationship with others (Cl1), not expressing trust and not encouraging the independent identity of others (Cl3, Cl4), on whom they themselves are dependent and by whom they are conditioned (Cl1, Cl2, Cl 3). They are likely to have difficulties in interpersonal relationships and in achieving true intimacy, even if desired, due to their poor expression of emotions and needs, poor spontaneity (Cl 1, Cl2, Cl3) and the tendency to judge themselves and others (Cl6). They do not always actively help or are close to the other person (Cl 2, Cl 3). However, these behaviors may include elements of neglect and forgetfulness of the needs of others (Cl 8).

Some personality traits of this profile such as low autonomy and self-affirmation, self-neglecting behaviors, interpersonal difficulties and depression, that were associated with the onset of T2-DM from this study, correspond to those emerging from other studies on personality and temperament and character in T2-DM patients. In fact in some studies T2-DM patients are described with the following dimensions of temperament and character: “inhibition of behavior and association with worry and pessimism, thus serving as an index of caution and a search for safety or optimism with overconfidence, a preference for shyness and quiet inactivity o active stimulation, and the need for a long or short time for adaptation (HA); (low NS) low desire and acceptance of new stimuli, and preference for repetition, impulsiveness or calm and stoic behavior; pattern (low NS/ high HA/ low RD), resistance in habitual styles and lack of flexibility, resistance to change and lack of cooperation (C). This pattern consists of weak objective-directed behavioral control indicated by the low self-directness (SD) score, low cooperativeness (C), poor ability to describe feelings, high anxiety and depression, worry (Harm-Avoidance. HA), and easily feeling fatigued [9,11,17,18,47,48].

Other studies have confirmed the adverse effects of the Type-D personality construct not only on medical aspects but also on clinically important psychological factors (i.e., depression, anxiety, and anhedonia) [49].

The Type D personality behaviors of avoiding expressing emotions in social interactions due to fear of social rejection or any other kind of disapproval from people may be described in SASB -Model, as behaviors of low autonomy and dependence on others with the consequence of having a perceived lack of social support. One study adds evidence that depression is associated with a higher risk for developing T2DM, and the association is stronger among people with severe depression [29,34,35].

Some authors have stated that it is clinically relevant to detect Type-D personality factors, i.e., negative affectivity and social inhibition, in order to prevent potential risk consequences such as medical complications (i.e., neuropathy, nephropathy, retinopathy, glaucoma, hypertension, other macro-, and micro-cardiovascular problems). Diabetic patients with Type-D personality showed more difficulties in realizing self-health management behaviors [6,8,16,17,34,46–50].

In conclusion, some personality factors described by other studies generally coincide with those that emerged from our work and these traits could pre-exist and be associated with the onset of diabetes, as shown in our study.

Our findings that personality traits with “Low Affiliation & Autonomy” were associated with the diabetes onset of 66.8% give rise to some considerations on which primary multidimensional prevention can be implemented.”

Which kind of prevention? The intervention that we hypothesize should be multidisciplinary, and take into account the other risk factors such, i.e., incorrect style of life, incorrect diet and should include the following points: nutrition education; psycho-educational and psychotherapeutic intervention and change of incorrect/unhealthy style of life [46,49–54]. Some studies showed that T2-DM patients might benefit from psycho-educational interventions, such as supportive psychotherapy, or other psychotherapeutic approaches (i.e., Holistic Psychotherapy; Mind- Body therapy, Behavioral CognitiveTherapy, Mindfulness Based Interventions) [51–57] which are designed to help patients change their personalities when coping with the symptoms of their illness: we suggest that these interventions could also be implemented in programs of primary prevention based on the screening of subjects with such personality traits.

In conclusion, our findings highlight that some traits of personality play an important role in the likelihood of diabetes onset, suggesting that systematic assessment and treatment of maladaptive intrapsychic behaviors could contribute to the prevention of diabetes in the general population and in high-risk individuals. On the basis of the personality traits that emerged, it can be hypothesized that in the context of primary prevention of diabetes, the multidisciplinary approach that takes into account both clinical and psychological variables would be the most suitable one.

## Limitations

Our study had the following limitation: a sampling bias was present in the data because all the subjects attended two institutions in the same city and thus might not be representative of healthy subjects and diabetic patients in general.

## Supporting information

S1 Appendix SASB—Structural Analysis of Social Behaviours: Rules for determining intrapsychic behaviours. (DOC)

S1 Table Cluster K-Means analysis. (DOC)

S1 Data Cluster K-Means analysis. (XLSX)
