# Supplementary material for: Structural analysis of social behavior: Using cluster analysis to examine personality profile associated with diabetes onset
Source: PLoS One. 2025 May 9;20(5):e0315895. doi: 10.1371/journal.pone.0315895 (PMC12063907; doi:10.1371/journal.pone.0315895)
Supplement: S1 Table — (DOC) [file pone.0315895.s002.doc]

**Table S1.** Cluster K-Means analysis. SASB Personality Profiles

| **Variable** | **Final Cluster Centers** | | **F** | ***P*** |
| --- | --- | --- | --- | --- |
|  | **Profile 1**  *Low Affiliation and Self-care*  *N* = 391 | **Profile 2**  *Low Affiliation and Autonomy*  *N* = 124 |  |  |
| SASB CL 1 | 4 | 4 | 17,749 | < 0.001 |
| SASB CL 2 | 7 | 4 | 331,833 | < 0.001 |
| SASB CL 3 | 7 | 4 | 324,593 | < 0.001 |
| SASB CL 4 | 6 | 5 | 83,120 | < 0.001 |
| SASB CL 5 | 5 | 5 | 2,421 | 0.120 |
| SASB CL 6 | 1 | 3 | 327,835 | < 0.001 |
| SASB CL 7 | 1 | 3 | 313,956 | < 0.001 |
| SASB CL 8 | 2 | 4 | 194,273 | < 0.001 |
